# Investigation of the Microstructure, Mechanical, and Wear Characteristics of the Boronized Layer on IN718 Alloy Treated Under Aging Conditions

**DOI:** 10.3390/ma19081531

**Published:** 2026-04-11

**Authors:** Lu Xu, Jinyu Quan, Xinyu Zhang, Rongwang Xiao, Xiaoming Zong, Shuqun Chen

**Affiliations:** 1 China Helicopter Research and Development Institute, Jingdezhen 333001, China; xul028@avic.com (L.X.); zhangxy602@avic.com (X.Z.); 2 College of Materials Science and Engineering, Beijing University of Technology, Beijing 100124, China; quanjinyu@emails.bjut.edu.cn; 3 AECC Aero Science and Technology Co., Ltd., Chengdu 610503, China; 2595556qq@163.com; 4 Jonhon Optronic Technology Co., Ltd., Luoyang 471003, China

**Keywords:** IN718 alloy, boronizing, wear resistance

## Abstract

Boronizing treatment has gained significant attention for its effectiveness in enhancing the wear resistance of IN718 alloy. However, conventional boronizing temperatures for IN718 are higher than its aging temperature, which can disrupt the aged microstructure and lead to degradation of mechanical properties. In this study, pack boriding was performed on solution-treated IN718 alloy in the aged condition (760 °C for 10 h followed by 650 °C for 8 h). The microstructure, mechanical properties, and tribological behavior of the boride layer formed on IN718 were systematically investigated. The boride layer, with a thickness of approximately 15 μm, was found to be primarily composed of CrB, Cr_2_B, Ni_2_B, and Fe_3_Ni_20_B_6_ phases. Following boronizing treatment, the surface hardness of IN718 was enhanced from 278 HV_0.2_ to 1938 HV_0.2_, with a concurrent significant increase in the yield strength of the bulk specimen. When sliding against a 440C steel counterface, the specific wear rate of the boronized sample was reduced by a factor of 49, and the friction coefficient decreased to 70% of that of the substrate. The dominant wear mechanism shifted from adhesive, abrasive, and oxidative wear for the untreated IN718 to abrasive and oxidative wear for the boronized specimen.

## 1. Introduction

IN718 is a representative nickel-based superalloy, with Fe, Cr, Nb, Mo, Ti, and Al as its principal alloying elements [1]. It exhibits excellent corrosion resistance, reliable fatigue life, and structural stability at temperatures up to 650 °C [2,3,4], making it critical for demanding applications in aerospace, nuclear reactors [5], and turbine blades [6]. As a typical precipitation-hardened alloy [7], IN718 is typically strengthened through solution annealing followed by a two-step aging process [8]. The solution treatment temperature is usually set above the solvus temperature of undesirable phases, such as the Laves phase, to dissolve them and homogenize the alloying elements [9]. Subsequently, the ordered face-centered cubic γ′-Ni_3_(Al,Ti) and body-centered tetragonal γ″-Ni_3_Nb phases precipitate during aging, providing effective precipitation strengthening [10]. However, the relatively low hardness and poor wear resistance of IN718 often necessitate surface hardening to enhance its overall performance. 

Boronizing is an effective thermochemical process in which boron atoms diffuse into the alloy surface to form a hard layer of metallic borides [11]. Pack boronizing of IN718 has been extensively studied. Previous research indicates that the composition of the boronizing agent is the primary factor influencing the resulting surface layer structure. Conventional boronizing agents typically consist of B_4_C (boron source), KBF_4_ (activator), and SiC (diluent) [12]. However, using SiC-containing agents can lead to the formation of a porous nickel silicide layer (e.g., Ni_2_Si, Ni_3_Si) on nickel alloys [13]. This silicide layer impedes the growth of the boride layer, and its comparatively low hardness (~500 HV) [14] compromises the wear resistance of the boronized surface. Consequently, SiC-free boronizing agents, such as EKABOR^®^ Ni, have been developed specifically for nickel-based alloys [15].

The tribological properties of boronized IN718 have also been a focus of research. I. Campos-Silva et al. [16] employed a boronizing agent containing 90 wt% B_4_C and 10 wt% KBF_4_ at 1000 °C for 6 h, producing a boride layer approximately 50 μm thick and consisting of mixed boride phases such as Ni_2_B, Ni_3_B, Ni_4_B_3_, and Cr_2_B. The surface hardness reached 25 GPa, about five times greater than that of the substrate. The minimum wear rate at room temperature (RT) was 3.1 × 10^−6^ mm^3^/N·m, roughly 63 times lower than that of the substrate (1.98 × 10^−4^ mm^3^/N·m). A. Günen et al. [17] reported that at 400 °C, the wear volume of the IN718 substrate was 8.1 times greater than that of the boronized IN718. Z. Wu et al. [18] also discovered that the boride layer on IN718 offers excellent wear resistance against Si_3_N_4_ ceramic balls at RT and 500 °C. At room temperature, the formation of a dense boron oxide tribolayer significantly reduced the wear rate to 1.81 × 10^−6^ mm^3^/N·m, an improvement of two orders of magnitude over the substrate (1.78 × 10^−4^ mm^3^/N·m). At 500 °C, the boronized sample exhibited a low wear rate of 3.24 × 10^−4^ mm^3^/N·m, with a metal oxide tribolayer providing an antifriction effect.

To the best of our knowledge, the boronizing temperature for nickel-based alloys is generally higher than 850 °C to obtain sufficient boride layer thickness. However, this temperature is substantially higher than the aging temperature of IN718 (720–760 °C), which compromises the aged microstructure and consequently degrades its mechanical properties. While the standard heat treatment after boronizing could restore the substrate structure [19,20], it significantly increases the process complexity and cost. Therefore, it is of great significance to combine the boriding and aging treatments together.

In the present work, pack boriding was performed on solution-treated IN718 alloy in the aged condition (760 °C for 10 h followed by 650 °C for 8 h). The phase structure of the boride layer was characterized using XRD and SEM. The mechanical and tribological properties were assessed by microhardness and friction tests. The findings of this work are expected to advance the application of boronizing treatment in nickel-based superalloys.

## 2. Experiments

**Figure 1:**
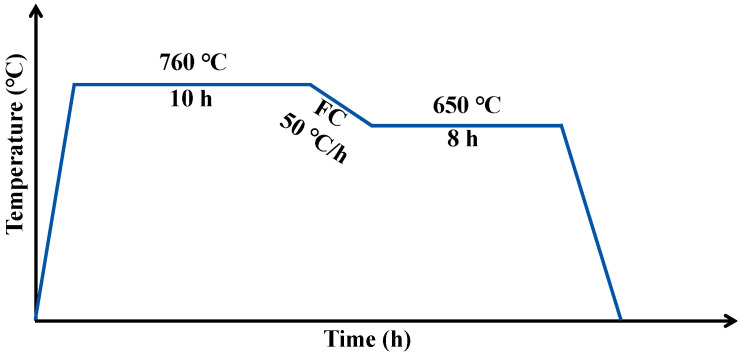
Schematic illustration of the boronizing process with an in situ aging process.

In this study, the base material was an IN718 alloy wrought bar subjected to solution treatment at 1050 °C for 1 h. The nominal composition of the IN718 is illustrated in Table 1. Powder-pack boronizing treatment was performed on IN718 alloy with a dimension of Φ 30 × 2 mm. Before boronizing, all sample surfaces were ground using 180 to 1000 grit SiC papers, followed by ultrasonic cleaning in ethanol for 10 min. The boronizing treatment was performed in a solid powder mixture consisting of 85 wt% B_4_C, 10 wt% KBF_4_, and 5 wt% amorphous boron. The samples were heated to 760 °C for 10 h, followed by cooling to 650 °C, held at this temperature for another 8 h, and then cooled to room temperature (one of the two double-age conditions recommended for IN718 according to the ASM Handbook [21]). A schematic illustration of the heat treatment cycle is shown in Figure 1. The bording process was carried out in a tube furnace system (NBD-O1200, NBD Ltd., Zhengzhou, China) under flowing argon.

The boronized IN718 was fixed in acrylic, ground using 180 to 3000 grit SiC papers, then polished using diamond paste (2.5 μm) to obtain a mirror cross-section. Scanning Electron Microscope (SEM, JEOL JSM 7200F, Tokyo, Japan) and energy-dispersive X-ray spectroscopy (EDS, Oxford X-Max, Oxford, UK) were applied to confirm the morphology and element distribution of the boride layer. The thickness of the boride layer was determined by averaging 10 measurements. The Focused Ion Beam (FIB, FEI Helios Nanolab 600i, FEI, Hillsboro, OR, USA) system was applied to cut the cross-sectional samples into foil. Imaging and selected-area electron diffraction (SAED) were analyzed via transmission electron microscope (STEM, FEI Talos, FEI, Hillsboro, OR, USA). The microstructure of the boride layer on alloy surfaces was characterized by X-ray diffraction (XRD, Bruker D8 Advance, Billerica, MA, USA). The hardness of the coatings was measured using a Vickers hardness tester (INNOVATEST Falcin 500, INNOVATEST Europe B.V., Maastricht, The Netherlands) with loads of 200 gf and 500 gf. Rockwell-C indentation testing was conducted to assess the interfacial bonding state of the boride layer onto the metal substrate. During the indentation test, a load of 1471 N and a 10 s dwell time was applied to the specimen surface. The friction performance of the boride layer was evaluated by a CFT-1 tribo-tester at room temperature. Specific sliding test parameters are listed in Table 2. The wear volume was measured using a Talysurf CCI white light interference surface profiler (Talysurf, Leicester, UK). The wear rate was calculated by:

(1) $$K\;=\;\frac{V}{\mathrm{Pvt}}$$

where *K* is the wear rate (mm^3^/N·m), *V* is the wear volume (mm^3^), *P* is the normal load (N), *v* is the sliding speed (m/s), and *t* is the test duration (s). In addition, a compression test was conducted on the specimen using a universal electronic testing machine (WDW-100) at a compression rate of 0.5 mm/min to evaluate the effect of aging treatment on the bulk specimen properties.

## 3. Results and Discussion

**Figure 2:**
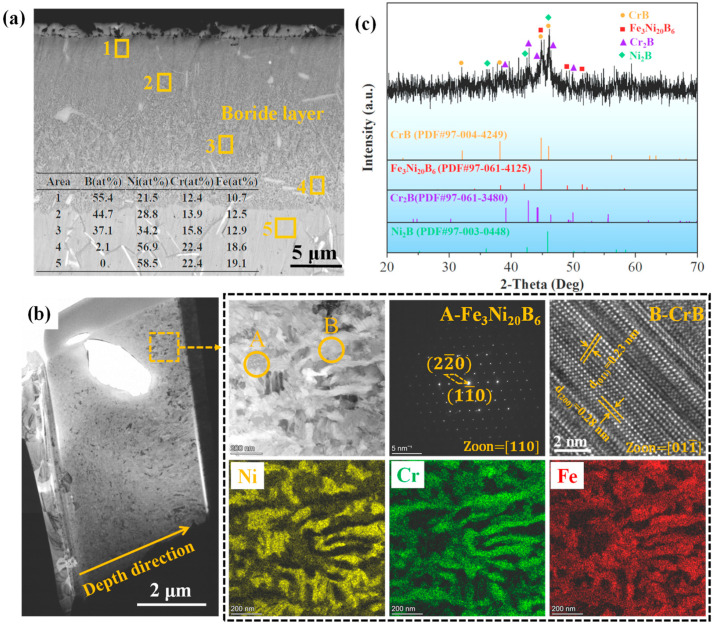
Cross-sectional SEM image (**a**), TEM images, EDS mappings and SAED patterns (**b**), and XRD pattern (**c**) of the boronized layer on IN718 alloy.

The cross-sectional SEM image of the boronized layer on IN718 is illustrated in Figure 2a. It can be observed that the boride layer is dense and compact, with a total thickness of ~15 µm. EDS analysis reveals that the boride layer is enriched with B, Cr, Ni, and Fe, and the boron content decreases progressively from the top (55.4 at% in Area 1) to the bottom (2.1 at% in Area 4). This gradient in boron concentration indicates that the microstructure of the boride layer consists of mixed metal borides. Based on its elemental composition, Area 6 can be identified as the IN718 substrate, with rod-like δ phase precipitated along the grain boundaries. Notably, rod-shaped δ phase is also observed within the boride layer (see Figure 2a), indicating its high thermal stability during the boronizing process.

As a common catalyst in the boronizing process, KBF_4_ will react as follows when heated [22]. Firstly, when temperature reaches 530 °C, KF and BF_3_ are liberated:

(2) $${\mathrm{KBF}}_{4}\;\to {\;\mathrm{BF}}_{3}↑\;+\;\mathrm{KF}$$

The BF_3_ react with B_4_C, providing active boron and BF_2_:

(3) $${2\mathrm{BF}}_{3}\;+\;B_{4}C\;\to \;{3\mathrm{BF}}_{2}↑\;+\;3[B]\;+\;C\;$$

At high temperatures, the BF_2_ produced from Equation (3) may decompose into BF_3_ and active boron:

(4) $${3\mathrm{BF}}_{2}\to \;[B]\;+{\;2\mathrm{BF}}_{3}\;↑\;$$

Amorphous B will also release active boron atoms after heating:

(5) B(Amorphou) → [B]

The active boron atoms and BF_3_ generated via Equations (3)–(5) can react with the metal atoms in IN718 to form a metal boride layer. Under low boriding temperatures, the composition of the boronizing agent plays a decisive role in determining the thickness of the resulting boride layer. In a recent study by L. Guan et al. [23], a boride layer of only ~8 μm in thickness was produced on GH4169 alloy after boriding at 800 °C for 6 h using an agent of 84 wt% B_4_C, 6 wt% KBF_4_, and 10 wt% ZrO_2_. Here, despite the relatively low boriding temperature of 760 °C, the high concentrations of KBF_4_ and amorphous boron in the agent enabled a boride layer with the desired thickness (~15 µm) to be obtained. However, the boride layer obtained is thinner than that formed on IN718 at 760 °C using the boriding agent EKABOR^®^NI (~23 µm) [24].

Since the metal boride phases formed on the IN718 alloy are complex, TEM analysis was employed to examine their structural details. FIB-SEM technology was used to prepare a cross-sectional sample, as shown in Figure 2b, with the analysis region located approximately 5 μm beneath the surface (corresponding to Area 2 in Figure 2a). The boride layer in this region consists of nanoparticle strips, with a particle size of approximately 200 nm. The corresponding EDS mapping images indicate that the boride layer can be divided into a Ni-rich region and a Cr-rich region (boron was not detected due to the limitations of the TEM detector). SAED and HRTEM images from regions A and B further confirm that the phase in the Ni-rich region is Fe_3_Ni_20_B_6_, while the phase in the Cr-rich region is CrB. This suggests that the boride layer in the selected region is composed of mixed Fe_3_Ni_20_B_6_ and CrB grains. In addition to TEM, XRD was used to gather additional phase information from the boride layer. As shown in Figure 2c, distinct diffraction peaks at 37.34°, 44.86°, and 46.05° correspond to the (021), (111), and (130) crystal planes of CrB, respectively. The Fe_3_Ni_20_B_6_, Cr_2_B, and Ni_2_B phases are also identified in the diffraction pattern.

**Figure 3:**
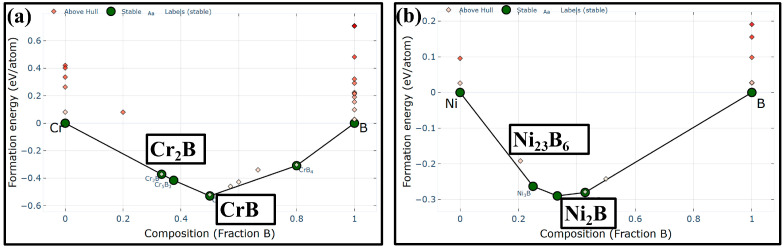
Formation energy curves of Cr-B atom-pairs (**a**) and Ni-B atom-pairs (**b**) derived from the Materials Project database [25].

The law of phase formation in the boronized layer can be explained by its formation energy. The calculated formation energy curves of Cr-B atom pairs and Ni-B atom pairs are shown in Figure 3 [25]. At moderate B content, CrB and Ni_2_B are preferentially formed, whereas Cr_2_B becomes more stable as the B content decreases. Ni_23_B_6_ is a metastable phase under low-B conditions, while Fe_3_Ni_20_B_6_ can be regarded as a derivative of Ni_23_B_6_ in which three Ni atoms are substituted by Fe. A relevant study proved that Fe doping can enhance the thermal stability of Ni_23_B_6_, thereby promoting its transition from a metastable to a stable phase [26].

**Figure 4:**
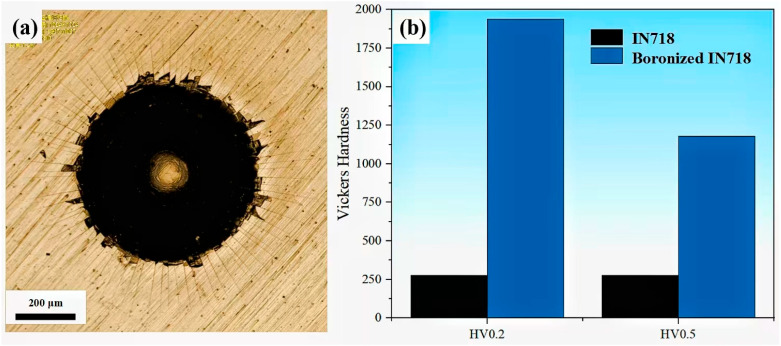
(**a**) Optical image of Rockwell C indent on boronized IN718; (**b**) A comparison of the surface hardness between IN718 and boronized IN718 alloys.

The bonding strength of the boride layer is a critical mechanical property that directly determines its service performance. Here, the interfacial bonding strength was evaluated using the Rockwell-C indentation method. The resulting indentation morphology is presented in Figure 4a. As observed, radial cracks and minor spalling are visible around the indentation, which corresponds to the HF2-HF3 level according to the VDI 3198 standard [27]. This indicates that the boride layer exhibits desired adhesion to the IN718 substrate.

The surface hardness test was performed on the untreated and boronized IN718, respectively. As shown in Figure 4b, when a 200gf test load is applied, the surface hardness of the boronized sample is 1938 HV_0.2_, which is about 6 times higher than that of the substrate (278 HV_0.2_). Based on the studies by M. Kulka et al. [28] and D.C. Lou et al. [29], the microhardness of the Cr-B phase (including Cr_2_B and CrB) and Ni-B phases (Ni_2_B) are within the range of 31.1 GPa and 10.8 GPa, respectively, demonstrating that the formation of chromium boride is a primary factor for surface hardening. In addition, when a 500 gf test load was applied, the surface hardness of the boronized IN718 decreased to 1179.3 HV_0.5_, which was due to the limited overall thickness of the boride layer. As the loading force increased, the boride layer was gradually penetrated, resulting in a reduction in the hardness value.

**Figure 5:**
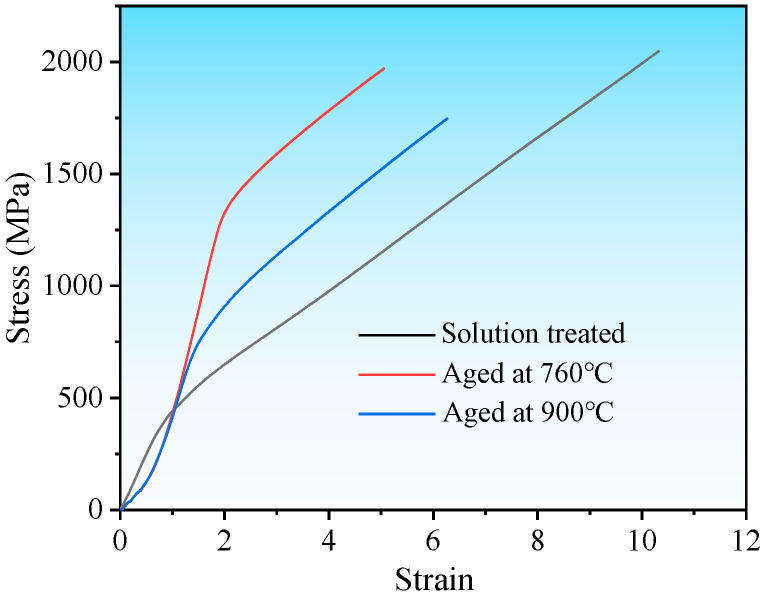
Compression curves of IN718 and aged IN718 alloys.

To evaluate the effect of aging treatment on the mechanical properties of bulk IN718 alloy, compression tests were performed on specimens both before and after aging. In addition to the specimen aged at 760 °C for 10 h and at 650 °C for 8 h, an aging treatment at 900 °C for 4 h (simulating the high-temperature boriding process for IN718) was also conducted for comparison. As shown in Figure 5, the compressive stress–strain curves reveal a significant difference between the three specimens. Specifically, the yield strength was 478 MPa for the solution-treated IN718, 1333 MPa for the specimen aged at 760 °C, and 716 MPa for the specimen aged at 900 °C. This clearly highlights the importance of selecting an appropriate temperature for the aging process. In IN718, precipitation strengthening serves as the primary strengthening mechanism [30]. The efficiency of this mechanism depends on the lattice coherence between the precipitate phase and the matrix. The two-step aging process promotes the precipitation of a high-volume fraction of γ′ and γ″ phases, which substantially enhances the mechanical performance [21].

**Figure 6:**
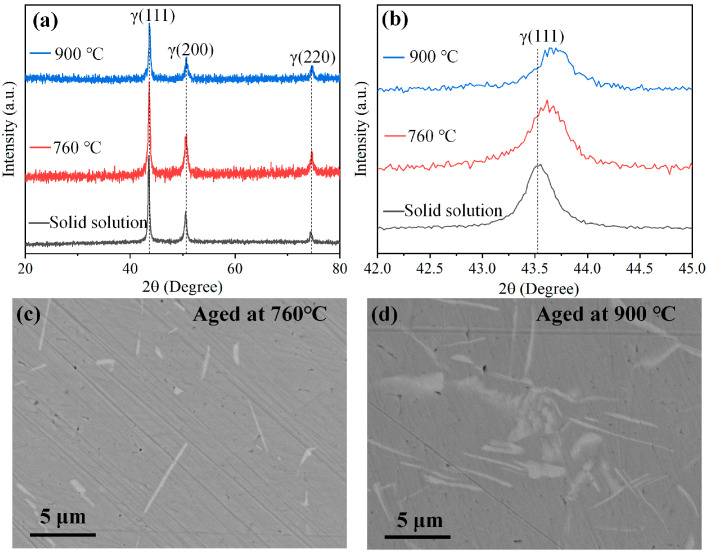
XRD patterns (**a**,**b**) and SEM images (**c**,**d**) of the studied IN718 alloys.

Figure 6a,b shows the XRD patterns of the IN718 alloy before and after two aging treatments. The diffraction peak at approximately 43.5° corresponds to the (111) plane of the γ phase. This peak shifts to higher angles after both aging treatments, indicating a decrease in interplanar spacing, as predicted by Bragg’s law. The observed peak shift is attributed to the precipitation of the γ′ and γ″ phases and the accompanying elemental redistribution [31]. Since both the γ′ phase—which has a face-centered cubic L12 crystal structure—and the γ″ phase—which has an ordered body-centered tetragonal Do22 crystal structure—are coherent with the γ matrix, they cannot be directly observed. During the aging process, elements such as Nb, Al, and Ti precipitate from the γ phase, leading to lattice contraction and a reduction in interplanar spacing [32]. Furthermore, Figure 6c,d shows that the higher heating temperature promotes the formation of rod-shaped δ phase precipitates, which are detrimental to the mechanical properties and result in a lower yield strength [33].

**Figure 7:**
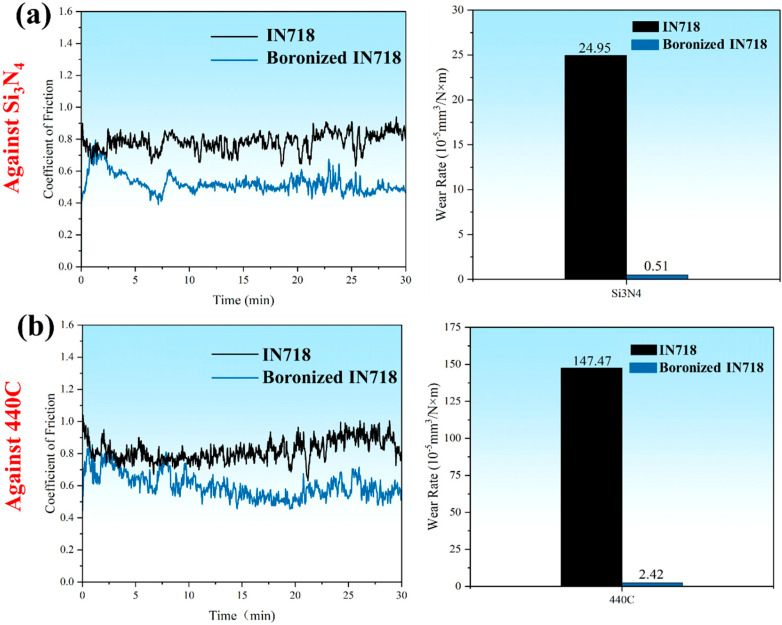
COF curves and wear rates of IN718 (**a**) and borionized IN718 (**b**) alloys under different friction conditions.

The tribological properties of the boronized IN718 and the untreated substrate were evaluated via reciprocating sliding tests under dry conditions. Figure 7 presents the corresponding friction coefficient curves and the calculated specific wear rates for tests against two different friction pairs. Overall, the boronizing treatment improves the tribological performance significantly. Firstly, it effectively reduces the friction coefficient. When tested against a 440C steel friction pair, the average coefficient of friction (COF) for the boronized IN718 is 0.6, approximately 70% of the substrate’s value (0.82). When testing against a Si_3_N_4_ friction pair, the average COF is reduced from 0.8 for the substrate to 0.52 for the boronized IN718, approximately 65% of the substrate’s value. Secondly, the treatment dramatically lowers the specific wear rate. Against the 440C friction pair, the wear rates are 5.1 × 10^−6^ mm^3^/N·m for the boronized IN718 and 2.5 × 10^−4^ mm^3^/N·m for the substrate, indicating a reduction by a factor of 49. Against the Si_3_N_4_ friction pair, the wear rates are 2.4 × 10^−5^ mm^3^/N·m and 1.5 × 10^−3^ mm^3^/N·m for the boronized and substrate samples, respectively, representing a reduction by a factor of 61.

**Figure 8:**
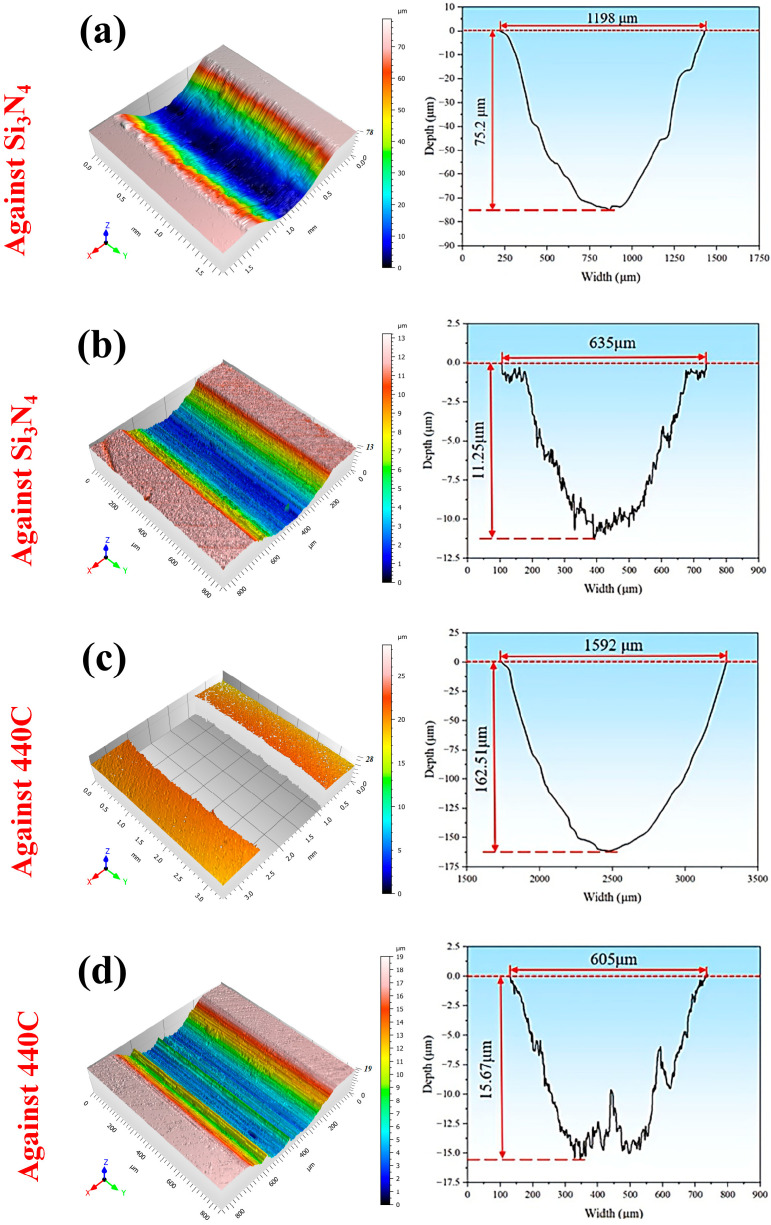
Wear morphologies and profiles of IN718 and boronized IN718 alloys under different friction conditions: (**a**) IN718 against Si_3_N_4_ ball; (**b**) boronized IN718 against Si_3_N_4_ ball; (**c**) IN718 against 440C ball; (**d**) boronized IN718 against 440C ball.

The morphologies and cross-sectional profiles of the wear scars are presented in Figure 8a–d. When sliding against the Si_3_N_4_ ball, the wear scar on the IN718 substrate exhibits a width and depth of 1198 µm and 75.2 µm, respectively. These values are 1.9 and 6.7 times greater than those measured on the boronized IN718. A much more severe wear occurs under the 440C counterbody condition. The substrate shows a maximum scar width of 1592 μm and a maximum depth of 162.5 μm, which are approximately 2.6 and 10.4 times greater than those of the boronized specimen, respectively. Notably, the substantial wear depth prevented the acquisition of a valid white light interference image (Figure 8c); therefore, the wear profile was measured using the contact probe method. Furthermore, the distinct grooves that appear on the wear scars prove that significant abrasive wear occurred.

**Figure 9:**
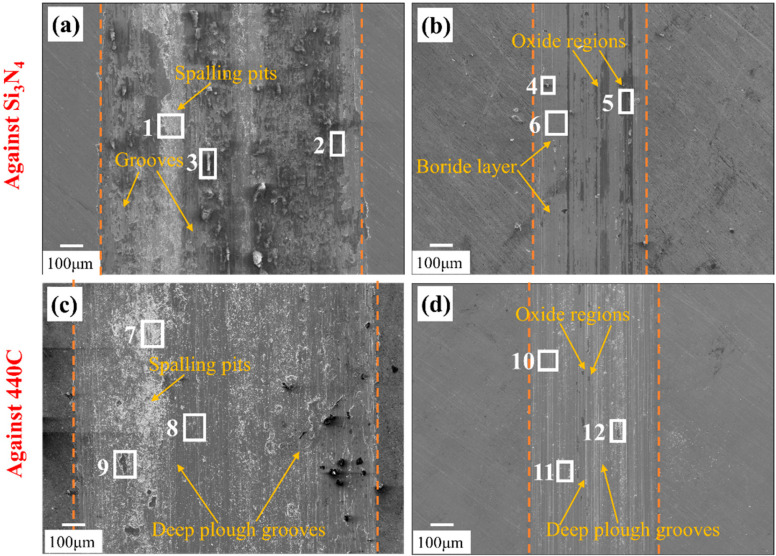
SEM images of the wear surfaces of IN718 and boronized IN718 alloys under different friction conditions: (**a**) IN718 against Si_3_N_4_ ball; (**b**) boronized IN718 against Si_3_N_4_ ball; (**c**) IN718 against 440C ball; (**d**) boronized IN718 against 440C ball.

The wear mechanisms of the untreated and boronized IN718 alloys were further elucidated through SEM and EDS analyses. Figure 9a–d presents the surface morphologies of the wear tracks concerned. When sliding against Si_3_N_4_, the wear surface of IN718 exhibits characteristics of severe oxidative and adhesive wear. As shown in Figure 9a, the scar features plastic deformation and spalling, with wear debris accumulated in the pits, which exhibit a clear indicator of adhesive wear. EDS analysis results of selected areas (Area 1–3) in Table 3 reveals high concentrations of oxygen, nickel, chromium, and iron, confirming significant oxidative wear. During the friction and wear process, the interfacial temperature in the local contact area rises profoundly, leading to severe surface oxidation. As a result, the IN718 wear debris was oxidized into Ni-Cr-Fe oxides quickly, which were gradually compacted to a tribolayer under the friction force. However, numerous microcracks are observed within the tribolayer, indicating that it fails to function in a protective role. Instead, it promotes the spallation of Ni-Cr-Fe oxides under cyclic stress, leading to significant fluctuations in the COF.

In contrast, for the boronized IN718 specimen, the dominant wear mechanisms transition to mild abrasive and oxidative wear. The wear scar (Figure 9b) is notably smoother and extends with few grooves, exhibiting the characteristics of abrasive wear. Meanwhile, certain large debris (Area 4) and a compact tribolayer (Area 5) can be identified on the wear scar. EDS analysis confirms this tribolayer is rich in oxygen and metallic elements, indicating that oxidative wear occurred. Crucially, a smooth region (Area 6) on the scar shows a high boron content (21.6 at%) with low oxygen (3.2 at%), demonstrating that the underlying boride layer remained largely intact and protective during the test.

When a 440C ball was employed, the wear surface of IN718 revealed severe oxidative, abrasive, and adhesive wear. As shown in Figure 9c, the wear scar contains substantial fine debris and deep grooves aligned with the sliding direction, exhibiting the characteristics of abrasive wear. The presence of spalling further confirms that adhesive wear occurred. EDS analysis reveals that the wear debris originates both from 440C steel and the IN718 substrate (for example, Area 8 contains 29.6 at% Fe over 18.7 at% Ni). Under cyclic stress, the wear debris is progressively crushed and oxidized, forming mixed Fe-Cr-Ni oxide debris, which displays the characteristics of oxidative wear. The COF fluctuates continuously as the resulting debris and oxides are repeatedly crushed by the 440C friction pair.

Under the sliding condition of the boronized IN718 against 440C, the wear mechanism shifts to abrasive wear and oxidative wear. As shown in Figure 8d, the wear scar on the boronized sample is smoother and exhibits grooves, characteristic of abrasive wear. Fine oxidized debris is observed to accumulate on the wear scar surface (Area 12). The smooth region (Area 10) shows high contents of B and Ni (25.1 at% and 43.0 at%, respectively) and a very low oxygen content (0.9 at%), indicating that the boride layer underwent only slight oxidation.

**Figure 10:**
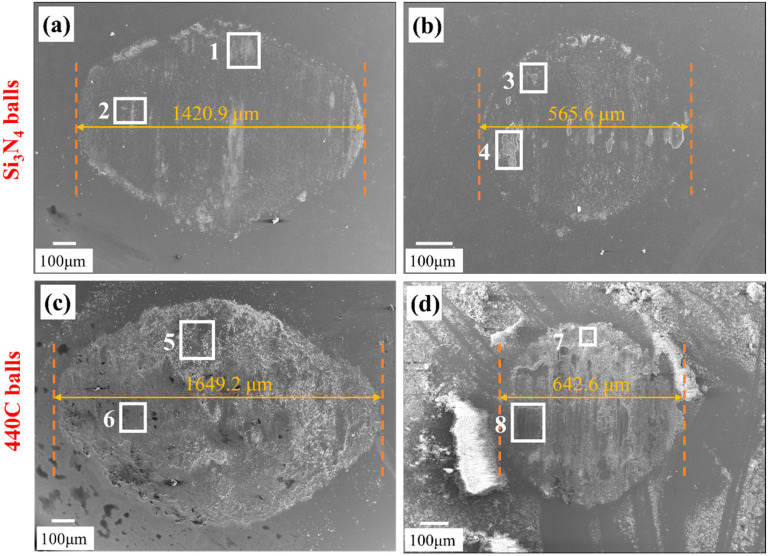
SEM images of the wear surfaces of friction balls under different friction conditions: (**a**) Si_3_N_4_ ball against IN718; (**b**) Si_3_N_4_ ball against boronized IN718; (**c**) 440C ball against IN718; (**d**) 440C ball against boronized IN718.

Figure 10a–d depicts the wear tracks on the Si_3_N_4_ and 440C balls after sliding tests. It is seen that the wear morphologies are distinct between the two counterbodies. Specifically, the wear tracks on the Si_3_N_4_ balls (Figure 10a,b) appear relatively smooth, with minimal adhered debris and a tribolayer. EDS analysis results of Areas 1 and 2 in Table 4 confirms the presence of oxygen, nickel, chromium, and iron, indicating the formation of mixed metal oxides transferred from the IN718. Similar findings are observed in Area 3 following sliding against the boronized IN718. However, a considerable amount of boron is detected in Area 4, suggesting that the tribolayer consists of a mixture of boron oxide and metal oxides. By contrast, the wear tracks on the 440C balls (Figure 10c,d) are extensively covered with adhered debris and a tribolayer. EDS results from Areas 5 and 6 indicate that the tribolayer formed on the ball surface is enriched in Fe, Ni, and Cr, with only a limited oxygen content. The high concentration of Ni suggests that significant material transfer (adhesive wear) occurred from the IN718 substrate to the 440C ball. When sliding against the boronized IN718, the wear debris (Area 7) is primarily composed of mixed Fe–Ni–Cr oxides, derived from both the 440C ball and boronized IN718 specimens. The tribolayer (Area 8) formed on the 440C ball consists of boron oxide and metal oxides, but with a limited area.

Based on the above observations, it is evident that the use of a 440C steel counterface induces more severe wear in both the untreated and boronized IN718 specimens. According to Wen Shizhu et al. [34], friction pairs composed of dissimilar metals are more prone to adhesive wear than those involving non-metallic materials, and the severity of adhesive wear tends to increase with the plasticity of the materials involved. Since both 440C steel and IN718 are metallic and exhibit considerable plasticity, their sliding contact readily promotes adhesive wear, leading to substantial material loss from the IN718 specimen. Concurrently, frictional heat generated during sliding promotes surface oxidation, producing abrasive metal oxide particles that further contribute to wear through abrasion. The boronizing treatment subsequently alters the wear mechanism. After boronizing, the surface of IN718 consists of high-hardness ceramic phases. Given that the hardness of 440C steel is only ~820 HV [35], the 440C counterface is likely to experience severe wear during the initial stage of sliding, generating a large amount of iron oxide debris (Fe_2_O_3_, with a hardness of approximately 1300 HV [36]). These hard oxide particles act as abrasives, grooving both the boride layer and the 440C ball. This abrasive action increases the contact area and promotes the generation of additional oxide debris, such as nickel oxide and chromium oxide. Furthermore, the metallic oxide debris fails to form a dense and continuous tribolayer on the surface of the friction ball. Consequently, the wear rate of the boronized IN718 alloy tested against the 440C counterface is higher than that observed with the Si_3_N_4_ counterpart.

## 4. Conclusions

In this work, the solution-treated IN718 alloy was subjected to pack boronizing under an aging condition. The microstructure and friction properties of the boride layer were systematically studied. The main findings are listed as follows:

(1) The boride layer formed on the IN718 alloy was dense and well-adhered, with a thickness of approximately 15 μm. Phase analysis revealed that the layer consists primarily of CrB, Cr_2_B, Ni_2_B, and Fe_3_Ni_20_B_6_.
(2) The surface hardness of the IN718 alloy was increased from 278 HV_0.2_ to 1938 HV_0.2_ after boronzing treatment. The concurrent aging treatment also significantly enhanced the yield strength of the bulk IN718 specimen.
(3) When sliding against a 440C steel counterface, the specific wear rate of the boronized sample was reduced by a factor of 49, and the friction coefficient decreased to 70% of that of the substrate. The dominant wear mechanism shifted from adhesive, abrasive, and oxidative wear for the untreated IN718 to abrasive and oxidative wear for the boronized specimen.

## Figures and Tables

**Table 1:** Nominal composition of the IN718.

Element	C	Ni	Cr	Mo	Al	Ti	Nb	Fe
Content (wt%)	0.04	53	19	3	0.5	0.9	5	BAL

**Table 2:** Sliding test parameters.

Mode	Normal Load	Stroke Distance	Sliding Speed	Testing Duration	Friction-Pairs
Reciprocating sliding	10 N	5 mm	33.3mm/s	30 min	Φ 4 mm Si_3_N_4_ ball and 440C ball

**Table 3:** Chemical composition of selected positions in Figure 9 determined by EDS.

Area	B (at%)	O (at%)	Ni (at%)	Cr (at%)	Fe (at%)
1	-	37.1	36.0	14.4	12.5
2	-	56.2	25.2	10.1	8.5
3	-	52.6	27.4	11.0	9.0
4	-	24.5	43.6	18.4	13.5
5	-	62.4	20.3	9.7	7.6
6	21.6	3.2	43.6	18.3	13.3
7	-	34.9	30.9	13.9	20.3
8	-	39.3	18.7	12.4	29.6
9	-	3.1	56.2	21.8	18.9
10	25.1	0.9	43.0	17.7	13.3
11	16.2	58.2	13.4	6.2	6.0
12	12.8	53.7	18.7	7.8	7.0

**Table 4:** Chemical composition of selected positions in Figure 10 determined by EDS.

Area	B (at%)	O (at%)	Si (at%)	N (at%)	Ni (at%)	Cr (at%)	Fe (at%)
1	-	44.7	16.9	2.8	21.6	7.6	6.4
2	-	27.3	38.1	13.4	12.3	4.8	4.1
3	-	47.6	25.4	7.8	11.5	3.9	3.8
4	12.7	43.1	1.0	4.0	19.8	11.4	8.0
5	-	5.2	-	-	34.7	18.6	41.5
6	-	3.6	-	-	28.8	17.9	49.7
7	-	47.7	-	-	19.7	10.4	22.2
8	40.5	32.1	-	-	11.4	10.6	5.4

## Data Availability

The original contributions presented in this study are included in the article. Further inquiries can be directed to the corresponding authors.
